# Transglutaminases in Dysbiosis As Potential Environmental Drivers of Autoimmunity

**DOI:** 10.3389/fmicb.2017.00066

**Published:** 2017-01-24

**Authors:** Aaron Lerner, Rustam Aminov, Torsten Matthias

**Affiliations:** ^1^B. Rappaport School of Medicine, Technion – Israel Institute of TechnologyHaifa, Israel; ^2^AESKU.KIPP InstituteWendelsheim, Germany; ^3^Gastroenterology Division, School of Medicine and Dentistry, University of AberdeenAberdeen, UK

**Keywords:** microbiome, dysbiosis, intestine, microbial transglutaminases, posttranslational modification, autoimmune disease, environment

## Abstract

Protein-glutamine γ-glutamyltransferases (transglutaminases, Tgs) belong to the class of transferases. They catalyze the formation of an isopeptide bond between the acyl group at the end of the side chain of protein- or peptide-bound glutamine residues and the first order 𝜀-amine groups of protein- or peptide-bound lysine. The Tgs are considered to be universal protein cross-linkers, and they play an essential role in a number of human diseases. In this review, we discuss mainly the bacterial Tgs in terms of the functionality of the enzymes and a potential role they may play in bacterial survival. Since microbial transglutaminases (mTgs) are functionally similar to the human homologs, they may be involved in the human disease provocation. We suggest here a potential involvement of Tgs in the pathologies such as autoimmune diseases. In this hypothesis, the endogenous mTgs that are secreted by the gut microbiota, especially in a dysbiotic configuration, are potential drivers of systemic autoimmunity, via the enzymatic posttranslational modification of peptides in the gut lumen. These mTg activities directed toward cross-linking of naïve proteins can potentially generate neo-epitopes that are not only immunogenic but may also activate some immune response cascades leading to the pathological autoimmune processes.

## Introduction

Transglutaminases (EC 2.3.2.13) (Tg), i.e., protein-glutamine γ-glutamyltransferases, belong to the class of transferases. They catalyze the formation of an isopeptide bond between the acyl group at the end of the side chain of protein- or peptide-bound glutamine residues and the first order 𝜀-amine groups of protein- or peptide-bound lysine. The transfer of the acyl group onto the lysine residue bound in the polypeptide chain initiates the cross-linking process. Deamination occurs if there is an absence of free amine groups. In this case, water acts as an acyl acceptor. Both deamidation and transamidation reactions are the hallmark of post-translational modification of proteins (PTMP) performed by Tgs. Tgs include nine enzyme families and they actually form a superfamily the representatives of which can be found in all Archaea and in some Bacteria and Eukarya (Makarova et al., 1999). This enzymatic reaction results in a significant change of the physico-chemical properties of proteins affecting viscosity, thermal stability, elasticity, water holding capacity, binding ability, consistency, texture, and resilience (Kieliszek and Misiewicz, 2014; Lerner et al., 2016).

The tTg of the Eukarya is a pleiotropic enzyme, which is expressed ubiquitously and abundantly in different human tissues (Reif and Lerner, 2004). It is involved into a variety of cellular processes such as growth, differentiation, migration, signaling, cytoprotection, apoptosis, survival, wound healing, angiogenesis, inflammation, and autophagy (Lerner et al., 2015c). Anti-tTg antibodies are frequently detected in celiac disease, and they are implicated in the small intestine damage in this disease (Griffin et al., 2002; Lerner et al., 2015d).

Microbial transglutaminases are extracellular enzymes (Kieliszek and Misiewicz, 2014). The most studied is the Tg from a *Streptoverticillium* sp. Unlike the human transglutaminases, mTgs are calcium independent, have a single structural domain, have lower molecular mass, and have a different reactivity toward certain proteins in food. They display a limited sequence homology to human homologs; these characteristics allow modifying the functionality of proteins in food products (Lerner and Matthias, 2015a,c,e). The production and application of mTgs in food processing is rapidly growing, aided by the use of genetic engineering to improve the functionality and properties of the enzymes. The massive production and the widespread use of mTgs can be attributed to the ease of manufacturing and the stability over a broad range of pH (from 5 to 9) and temperatures, which are well within the range of physico-chemical conditions encountered in the human gastrointestinal tract (Yokoyama et al., 2004).

Transglutaminases from the Eukarya exhibit catalytic activities and biochemical properties similar to the mTgs, despite having a limited homology at the amino acid sequence level (Lerner and Matthias, 2015a). It has been suggested that all mTgs are proteases and that the eukaryotic Tgs have evolved from an ancestral protease (Makarova et al., 1999).

Microbial transglutaminases are used extensively as additives in food processing industry and the production; the biocatalytic properties and other industrial aspects of mTgs are covered well in a number of recent reviews (Yokoyama et al., 2004; Kieliszek and Misiewicz, 2014; Strop, 2014; Lerner and Matthias, 2015a,c,e). mTgs are considered, at least by producers, to be safe, non-toxic, non-allergenic, not immunogenic, and not pathogenic, The main aim of this review is to evaluate the potential effects that could be exerted by the mTgs in the human gastrointestinal tract following their ingestion with the industrially processed food. Another aspect of the review concerns the mTgs that are produced by the human gut microbiota. In particular, the diversity of mTgs present, the regulation of expression and secretion of mTgs, and the role of mTgs in the survival of gut microorganisms. We hypothesize that the presence of external mTgs in the gut as well as the changed profile of the internally produced mTgs as a result of dysbiotic conditions may potentially affect human health.

## Substrate Specificity of mTgs

Because of the importance of Tgs in normal physiology as well as in pathology, they have been extensively studied over the past six decades. The substrate specificity of enzymes in this class, however, remains largely elusive (Strop, 2014). The PTMP by formation of isopeptide bonds between the side chain of glutamine residues in a peptide or protein and different primary amines is the basis for the Tg functions. Generally, while Tgs are specific toward the acyl donor substrates, they are less selective toward the acyl acceptor substrates. It seems clear that the exposure of both Gln and Lys residues on the surface of a target protein is essential, but not sufficient for the effective Tg activity. Denatured, unfolded or fibrous peptides and proteins appear to be more preferred substrates for mTgs compared to the native or folded ones.

The most preferable glutamine donor substrates have been identified using phage display libraries (Rachel and Pelletier, 2013), but the characterization of the full substrate range, especially of the acyl acceptors, is still far from clear. With the use of proteinaceous protease inhibitors, Taguchi et al. (2000) have further defined the nature of the flanking amino acid residues that affect the mTg reactivity toward the substrate lysine residue. More recently, potential substrates for mTgs have been screened using a fluorescence-based array (Malešević et al., 2015). The strongest binding of the donor peptides was found in spots containing aromatic amino acids adjacent to the reactive lysine residue and for peptides containing two lysine residues (Malešević et al., 2015). Further refinements of the mTg acyl-acceptor substrate specificity have been obtained by Gundersen et al. (2014). They found that very short-chain alkyl-based amino acids such as glycine serve as acceptor substrates and that the esterified α-amino acids such as Thr, Ser, Cys, and Trp also display the reactivity. The authors corroborated the fact that a ring near the amine group, particularly aromatic, is favorable for the reactivity. In general, the amine groups that are bound to a less hindered carbon increase the reactivity. With the appropriate primary amines as spacers, various functional groups such as carboxyl groups, phosphate groups, saccharides, and others can be incorporated into peptides and proteins by the mTg activity.

It should be noted, however, that these studies have been performed *in vitro*, while there is deficiency of *in vivo* studies with the intestinal substrate repertoire and the mTgs of the human gut microbiota. A number of studies suggested the importance of mTgs for the survival of microorganisms and these are overviewed in the next section.

## mTgs Are Essential for the Survival of Microorganisms

The human gut microbiota plays an essential role in human health being involved in a number of functions including nutritional, metabolic, signaling, developmental, and immune processes in the human body (Marchesi et al., 2015). When the intricate balance of gut microbiota is disrupted as a consequence of environmental factors such as antibiotic usage, diet change, stress, infections, and injury or because of genetic predisposition, it results in dysbiotic conditions. Dysbiosis is associated with a broad range of human diseases including irritable bowel syndrome (IBS), inflammatory bowel disease (IBD), colorectal cancer, stomach ulcers and cancer, asthma, atopy, metabolic syndrome, diabetes, hypertension, autism, multiple sclerosis, and other pathologies (Petersen and Round, 2014; Parekh et al., 2015). Dysbiotic conditions are characterized by the loss of microbial diversity, the decrease of beneficial microorganisms, and the overgrowth of opportunistic pathogens (Petersen and Round, 2014). The latter group increases the overall community virulence, disables immune surveillance, and promotes an overall inflammatory response (Petersen and Round, 2014). Inflammation and dysbiosis bolster each other leading to the self-perpetuating process, with uncontrolled inflammation and tissue damage, which benefits the pathobiont population due to the production of nutritious proteinaceous substrates, derived from the inflamed tissue breakdown (Lamont and Hajishengallis, 2015; Zhu et al., 2015).

Recently it has been shown that a Tg may downregulate an antimicrobial peptide, thus protecting crabs from a bacterial pathogen (Rao and Mehta, 2004). In *Drosophila*, the Tg activity is essential for the entrapment of invading pathogens (Wang et al., 2010). Moreover, this activity is pivotal to the maintenance of the normal commensal microbiota (Shibata and Kawabata, 2015). Inhibition of this activity by RNAi leads to the overproduction of antimicrobial peptides in the gut, to the changes in the composition of gut microbiota and, subsequently, to the reduced life span of flies.

Representatives of all three domains of life possess a remarkable capability to survive under a variety of adverse environmental conditions. One of the important mechanisms of protection from the environmental stresses is building a rigid and biochemically inert boundaries between the environment and cellular content. These structures are highly stable and resistant to various environmental insults. They can be very different at biochemical level, composed of peptidoglycan in the case of the Bacteria, S-layers in the Archaea, cellulose in plants, and chitin in many representatives of animals and fungi. In mammals, humans included, the outermost protective physical barrier of the skin in the form a cornified cell envelope beneath the plasma membrane is formed due to the Tg activity linking the small proline-rich proteins produced. In insects, they are composed of complex carbohydrates such as chitin and the heavily cross-linked scaffold of proteins to form complex structures such as sheath, cuticle, and epicuticle.

The isopeptide bonds formed by the mTg are of a great physiological significance since once the bonds are formed, they cannot be hydrolyzed by any known eukaryotic enzyme, and they also exhibit a formidable resistance toward reducing agents, detergents, and chaotropic agents. These protective structures, synthesized by microorganisms using mTg, therefore, may contribute to a better survival of microorganisms in hostile environments, including the gut with the excretion into the gut lumen of bile acids, digestive enzymes, antimicrobial peptides, and immunoglobulins (Rao and Mehta, 2004). It is not entirely clear, however, what is the range of diversity and what are the quantities of mTgs produced in the gut. Some of this fragmented information is summarized in the next section.

## mTgs Are Expressed and Secreted Into the Human Intestinal Lumen

Based on the protective mechanisms of the host to keep the bacteria at bay, it is logical to assume that the microbial protective mechanisms, including the synthesis of mTgs, should be abundant. Indeed, the similarity searches (Altschul et al., 1990) among the genomes and metagenomes of human intestinal microbiota produces multiple hits with mTg, and Supplementary Table S1, summarizes the first 100 of them, with taxonomic affiliation of these mTgs-encoding bacteria, including the genus and, if available, species designation. Interestingly, the vast majority of bacteria possessing the corresponding genes belong to the Firmicutes phylum, one of the two most prevalent phyla in the gut, which is dominated by this and the Bacteroidetes phyla. The only representatives of other taxonomic groups in **Table 1**, which is highly biased because mostly the industrial producer strains are presented, lists just a few of the Enterobacteriales. The vast majority is represented by the Actinobacteria and, to a much lesser extent, some corynebacteria and bacilli. Again, no Bacteroidetes representatives are encountered in this list as well. The significance of this discovery cannot be fully appreciated at the moment and provokes further thoughts on the particular strategies employed by different groups of bacteria in their interaction with the host.

**Table 1:** Bacterial strains used in the food industry for mTg production and the corresponding enzyme yields (adapted from Kieliszek and Misiewicz, 2014; Liu et al., 2014; Salis et al., 2015).

Strain	Yield
*Actinomadura* sp. T–2	n/a^*a*^
*Bacillus circulans* BL32	0.28 U/ml
*Bacillus subtilis*	n/a
*Corynebacterium ammoniagenes*	n/a
*Corynebacterium glutamicum*	n/a
*Enterobacter* sp. C2361	0.77 U/ml
*Providencia* sp. C1112	0.92 U/ml
*Streptoverticillium mobaraense*	0.9–3.4 U/ml
*Streptomyces platensis* M5218	0.66 U/ml
*Streptomyces hygroscopicus*	n/a
*Streptomyces lividans*	n/a
*Streptomyces lividans* JT46/pAE053	2.2 U/ml
*Streptomyces lydicus*	1.3 U/ml
*Streptomyces platensis*	1.4 U/ml
*Streptomyces sioyaensis*	3.3 U/ml
*Streptoverticillium griseocarneum*	1.46 U/ml
*Streptoverticillium ladakanum* NRRL–3191	0.28–1.55 U/ml
*Streptoverticillium* sp. s–8112	1.46 U/ml
*Escherichia coli* NP650C001	15,1 U/mg
*Escherichia coli* NP668C003	24 U/mg

*^a^Data not available.*

Although the present review deals mainly with the mTg-producing microbiota of the human gut, it is important to mention here that there is an additional source of mTgs continuously coming with the diet. These are recombinant mTgs that are used extensively by food processing industries, and mTgs remain in the processed food to enter the gastrointestinal tract (Lerner and Matthias, 2015a,e). As mentioned before, **Table 1** is the summary of strains, including recombinant *E. coli*, used by the food industry to produce these enzymes, with the corresponding yields (Kieliszek and Misiewicz, 2014; Liu et al., 2014; Salis et al., 2015). The conclusions that can be drawn from the data presented in these two tables are:

(1) The mTg genes are encoded in the genomes of many intestinal bacteria but almost all of them are the Firmicutes. This suggests the importance of this activity for the phylum representatives for the survival and persistence in the gastrointestinal tract.
(2) The industrially engineered and produced mTgs are from the bacteria that are not the integral part of the normal gastrointestinal tract and, therefore, may have different properties than the endogenously produced enzymes.
(3) The additional supply of recombinant mTgs via the processed food increases the luminal concentrations of mTgs, thus exerting an additive or potentiating effect on the existing endogenous enzyme equilibrium in the human gut lumen.
(4) The consumption of probiotics is increasing worldwide. It is estimated the global probiotics demand was worth USD 27.9 billion in 2011 and is expected to reach USD 44.9 billion in 2018 (Transparency Market Research, 2013). Probiotics Market (Dietary Supplements, Animal Feed, Foods and Beverages) – Global Industry Size, Share, Trends, Analysis, Growth and Forecast 2012 – 2018^1^. The vast majority of the probiotics used belong to the *Firmicutes* and *Actinobacteria*, which possess the mTg-encoding genes. These may represent an additional substantial source of mTgs entering the gut lumen.
(5) The consequences of elevated concentrations of mTgs are not clear but the Tg activities in general have been linked to fibrosis, Huntington’s disease, celiac disease, and cancer. The serious physiological disorders have prompted the development of Tg inhibitors (Keillor and Apperley, 2016).

It is not possible to estimate accurately the level of various mTgs as it is greatly dependent on the indigenous production and the composition of gut microbiota (Bacteroidetes/Firmicutes ratio) as well as the external supply via diet (industrially processed food) or probiotic consumption. Very approximate estimates can be made as follows:

(1) Each kilogram of food treated with mTg contains about 50–100 mg of the residual mTgs (Malandain, 2005).
(2) Examples of the mean mTg yield by bacteria used in industrial mTg production (Table No 2), (Kieliszek and Misiewicz, 2014; Liu et al., 2014; Strop, 2014; Salis et al., 2015) are *Streptoverticillium* sp. 1.43 unit/ml, *Streptoverticillium mobaraense* (ajinomoto “wild type”) 1.25 unit/ml or 22 unit/mg and the yield in recombinant *Escherichia coli* producer may reach 19.55 unit/mg.
(3) No information, to our knowledge, is available on the amount of mTgs secreted into the human gut lumen. The production by the host, however, is detectable, with the Tgase activity being described for the human jejunum (Bruce et al., 1985).
(4) As a result of the increased use of mTgs in food processing industry, the standard Western diet now contains large amounts of mTgases (Kieliszek and Misiewicz, 2014; Lerner and Matthias, 2015e), with a maximum daily intake reaching up to 15 mg.
(5) Assuming the additive effect of the daily intake of 10^9^ viable cells of *Bifidobacterium bifidum, B. longum, B. adolescentis*, or *B. infantis* taken as probiotics, and which secrete mTgs (Kailasapathy and Chin, 2000) to the 100 trillion of bacteria constituting the human microbiome (with their own mTgs load), there is a substantial and continuous presence of mTg activity in the human intestinal lumen. Interestingly, a new application for mTg crosslinking have been proposed that can be used as a new probiotic carrier for the enhanced gastrointestinal transit and storage (Yew et al., 2011).

What could be the potential effects of the endogenous and exogenous mTgs present in the gut milieu? These potentials are briefly discussed in the next section.

## mTg and Intestinal PTMP

In celiac disease, the autoantigen is tTg, which is capable of deamidating or transamidating gliadin (Reif and Lerner, 2004; Lerner et al., 2015c). This PTMP occurs below the epithelium, resulting in neo-epitopes of gliadin docked on the tTg, inducing anti-tTg or anti neo-epitope tTg autoantibodies (Lerner et al., 2015a). These are the well-known serological markers of celiac disease (Lerner, 2014; Lerner et al., 2015b). Recently it has been shown that another enzyme belonging to the tTg family of proteins is a potent inducer of specific antibodies in celiac disease patients (Lerner and Matthias, 2015d). This enzyme belongs to the mTgs, which are extensively used in the food industry. Moreover, the same food additive has been suggested as a new environmental trigger and potential inducer of celiac disease (Lerner and Matthias, 2015a,c,e).

The prominent features of the mTgs encompass the multitude of functions and properties, which may contribute to the potential PTMP capabilities in the human gut lumen:

(I) mTgs display a limited specificity toward the natural and non-natural acyl-acceptor substrates, which broadens the range of modifications involving the Gln-containing peptides and proteins (Kieliszek and Misiewicz, 2014). This results in the modification of the protein conformation and other, more extensive conformational changes. The latter is due to the more relaxed bonding capabilities, which allow making bonds among the homologous and non-homologous proteins or peptides. Thus the high molecular weight conjugates can be formed, with different conformational, physical, electrical, chemical, and immunogenic properties (Carvajal et al., 2011; Lerner and Matthias, 2015d,e).
(II) Bonds formed by Tgs are resistant to proteolysis and considered to be irreversible. Thus, the capabilities for digestion of foreign or improperly folded proteins to eliminate them from the gut lumen can be compromised (Lerner and Matthias, 2015e; Lerner et al., 2015c).
(III) mTgs play a significant role in the survival of microorganisms (Rao and Mehta, 2004). Infections as well as the cross-linked nutritional constituents such as mTg-aided gluten derivatives can increase the gut permeability. This results in the leaky gut allowing more immunogenic foreign molecules to enter systemic circulation and induce strong immune responses, including celiac disease and other autoimmune diseases (Lerner and Matthias, 2015e).

In the light of the close functionality between the human and microbial Tgs and the involvement of the former in disease, it is possible to suggest the potential involvement of latter in human disease as well. Besides, the concentrations of mTgs achieved in the gut lumen are likely to be much higher thus increasing their pathogenic potential. As a consequence, the increase of total Tg in the lumen and the higher titers of anti-Tg antibodies achieved may contribute to more extensive tissue damage in the intestine. Further, we summarize the potential pathogenic role played by tTg in human autoimmune diseases.

## tTg Involvement in Autoimmune Diseases

Tissue transglutaminase is a pleiotropic enzyme expressed ubiquitously and abundantly. It has been implicated in a variety of physiological processes (Reif and Lerner, 2004; Lerner et al., 2015c). Aberrant activation or deregulation of its functions is involved in numerous human diseases. The best known is the celiac disease, but there are also extra intestinal entities. tTg plays a significant role in diseases of inflammatory, degenerative-age related, neurodegenerative, malignant, metabolic, hormonal, genetic, and autoimmune nature (Lerner et al., 2015c). A few examples to mention here are: type 1 diabetes, dermatitis herpetiformis, multiple sclerosis, SLE, bullous pemphigoid, Sjogren’s syndrome, and rheumatoid arthritis (Lerner et al., 2015c).

As members of the Tg family of proteins, the mTgs behave similarly. Thus, they are capable of reaching the similar levels of PTMP, and they may even have a higher immunogenic potential compared to the human counterpart. They possess similar epitopes and, when probed with the sera of the celiac disease patients, there is a significant cross-reactivity. Thus the anti tTg autoantibodies from celiac disease patients react to and display a significant level of anti-mTg antibodies in the sera (Lerner and Matthias, 2015d). Based on all the evidences presented, mTgs may contribute to the induction, development, and perpetuation of the autoimmune processes.

## The Hypothesis: mTg is a Potential Driver of Systemic Autoimmunity

**FIGURE 1:**
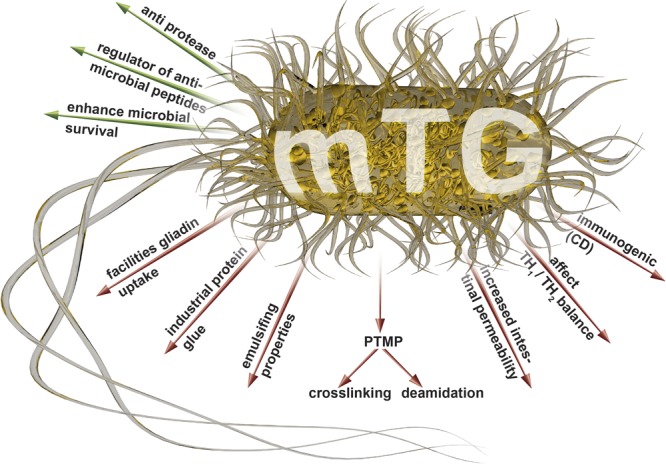
**Luminal (green arrows) and systemic (red arrows) effects of the mTgs that may drive autoimmunity (adapted from Griffin et al., 2002; Kieliszek and Misiewicz, 2014; Martins et al., 2014; Lerner and Matthias, 2015a,e; Lerner et al., 2016).** mTg, microbial transglutaminase; PTMP, post translational modification of proteins.

We hypothesize here that the endogenous mTgs, secreted by the gut microbiota, especially in a dysbiotic configuration (Firmicutes/Bacteroidetes ratio), is a potential driver of systemic autoimmunity, via the gut luminal PTMP. Additionally, the massive use of mTgs in processed food and the increased use of probiotics with mTg activities may be additional contributors to the enhanced luminal PTMP. **Figure 1** depicts the multiple intra and extra intestinal effects of the mTgs. Similarly to the tTg in the same protein family, they well-known PT protein modifiers. These enzymes are secreted by a variety of gut bacteria belonging to the Firmicutes phylum. Compared to the tTg, the microbial enzymes are less sensitive and specific to substrates, and they are calcium independent. They are also anti-proteases, possessing the emulsifying capacities, and they may increase the survival of bacteria (Martins et al., 2014; Lerner and Matthias, 2015b). All these capacities may contribute to the increase in the intestinal permeability thus potentiating immune and autoimmune responses.

## Summary

It is hypothesized here that the mTgs produced by the gut microbiota, especially in the dysbiotic configuration, are the potential drivers of systemic autoimmunity, via the PTMP activities in the gut lumen. In addition, the massive use of mTgs in the processed foods and the increasing use of probiotics with mTg activities may be the additional contributors to the enhanced luminal PTMP. The substantial luminal activity of the mTgs leading to cross-linking of naïve proteins can potentially generate neo-epitopes that are not only immunogenic but may also contribute to the activation of some undesirable inflammatory pathways involved in autoimmune processes.

It has been stated recently that the microbial enzymes are “completely safe to health. Furthermore, enzymes do not remain active in the final products” (Bueno et al., 2016). Several observations contest this declaration, as recently summarized (Lerner and Matthias, 2016a,b; Matthias et al., 2016). Notably, in celiac disease, mTg docked by gliadin is immunogenic, resulting in anti-neo epitope mTg (Matthias et al., 2016). If future research substantiates the present hypothesis, it will affect food product labeling, food additive policies in the food industry, and consumer health education.

## Author Contributions

AL and RA screened literature, analyzed the data, and wrote the manuscript. TM designed, edited and revised critically the manuscript.

## Conflict of Interest Statement

The authors declare that the research was conducted in the absence of any commercial or financial relationships that could be construed as a potential conflict of interest.

## Footnotes

1 http://www.transparencymarketresearch.com/probiotics-market.html

## Abbreviations

Tg: microbial transglutaminase
PTMP: post translational modification of proteins
Tg: transglutaminase
tTg: tissue transglutaminase
