# Supplementary material for: Transglutaminases in Dysbiosis As Potential Environmental Drivers of Autoimmunity
Source: Front Microbiol. 2017 Jan 24;8:66. doi: 10.3389/fmicb.2017.00066 (PMC5258703; doi:10.3389/fmicb.2017.00066)
Supplement: Supplementary file 1 [file Table_1.docx]

**Supplementary Material:**

**Table 1S. Taxonomic position of gut bacteria possessing the genes encoding mTgs.**

|  | **Source** | **Organism** | **Bacteria** |
| --- | --- | --- | --- |
| **1** | **Firmicutes bacterium CAG:555** | **Firmicutes bacterium CAG:555** |  |
| **2** | **Firmicutes bacterium CAG:240** | **Firmicutes bacterium CAG:240** |  |
| **3** | **Herbinix hemicellulosilytica** | **Herbinix hemicellulosilytica** | **Firmicutes; Clostridia; Clostridiales** |
| **4** | **[Clostridium] stercorarium** | **[Clostridium] stercorarium** | **Firmicutes; Clostridia; Clostridiales;** **Ruminococcaceae;**  **Ruminiclostridium** |
| **5** | **[Clostridium] cellulosi** | **[Clostridium] cellulosi** | **Firmicutes; Clostridia; Clostridiales;**  **Ruminococcaceae;**  **Ruminiclostridium.** |
| **6** | **Roseburia sp. CAG:303** | **Roseburia sp. CAG:303** | **Firmicutes; Clostridia; Clostridiales;**  **Lachnospiraceae;**  **Roseburia** |
| **7** | **Herbinix sp. SD1D** | **Herbinix sp. SD1D** | **Firmicutes; Clostridia; Clostridiales;**  **Lachnospiraceae;**  **Herbinix.** |
| **8** | **[Ruminococcus] torques** | **[Ruminococcus] torques** | **Firmicutes; Clostridia; Clostridiales;**  **Lachnospiraceae; Blautia.** |
| **9** | **Eubacterium rectale CAG:36** | **Eubacterium rectale CAG:36** | **; Firmicutes; Clostridia; Clostridiales; Eubacteriac**  **eae; Eubacterium** |
| **10** | **Clostridium sp. CAG:510** | **Clostridium sp. CAG:510** | **Firmicutes; Clostridia; Clostridiales; Clostridiac**  **eae; Clostridium** |
| **11** | **Eubacterium sp. CAG:248** | **Eubacterium sp. CAG:248** | **Firmicutes; Clostridia; Clostridiales; Eubacteriac**  **eae;**  **Eubacterium;** |
| **12** | **Firmicutes bacterium CAG:424** | **Firmicutes bacterium CAG:424** | **Firmicutes;** |
| **13** | **Lachnospiraceae bacterium NK4A136** | **Lachnospiraceae bacterium NK4A136** | **Firmicutes; Clostridia; Clostridiales;**  **Lachnospiraceae.** |
| **14** | **Lachnospiraceae bacterium AC2014** | **Lachnospiraceae bacterium AC2014** | **Firmicutes; Clostridia; Clostridiales;**  **Lachnospiraceae.** |
| **15** | **Lachnospiraceae bacterium NC2008** | **Lachnospiraceae bacterium NC2008** | **Firmicutes; Clostridia; Clostridiales;**  **Lachnospiraceae.** |
| **16** | **Pseudobutyrivibrio sp. LB2011** | **Pseudobutyrivibrio sp. LB2011** | **Firmicutes; Clostridia; Clostridiales; Lachnospiraceae; Pseudobutyrivibrio** |
| **17** | **Clostridium sp. CAG:448** | **Clostridium sp. CAG:448** | **; Firmicutes; Clostridia; Clostridiales; Clostridiac**  **eae; Clostridium** |
| **18** | **Lachnospiraceae bacterium A4** | **Lachnospiraceae bacterium A4** | **Firmicutes; Clostridia; Clostridiales;**  **Lachnospiraceae.** |
| **19** | **Eubacterium sp. CAG:38** | **Eubacterium sp. CAG:38** | **Firmicutes; Clostridia; Clostridiales; Eubacteriac** **eae; Eubacterium** |
| **20** | **Pseudobutyrivibrio ruminis** | **Pseudobutyrivibrio ruminis** | **Firmicutes; Clostridia; Clostridiales;Lachnospiraceae; Pseudobutyrivibrio.** |
| **21** | **Pseudobutyrivibrio sp. MD2005** | **Pseudobutyrivibrio sp. MD2005** | **; Firmicutes; Clostridia; Clostridiales;Lachnospiraceae; Pseudobutyrivibrio** |
| **22** | **Firmicutes bacterium CAG:272** | **Firmicutes bacterium CAG:272** | **Firmicutes;** |
| **23** | **Pseudobutyrivibrio ruminis** | **Pseudobutyrivibrio ruminis** | **Firmicutes; Clostridia; Clostridiales;Lachnospiraceae; Pseudobutyrivibrio** |
| **24** | **Lachnospiraceae bacterium AC2029** | **Lachnospiraceae bacterium AC2029** | **Firmicutes; Clostridia; Clostridiales; Lachnospiraceae** |
| **25** | **Pseudobutyrivibrio ruminis** | **Pseudobutyrivibrio ruminis** | **Firmicutes; Clostridia; Clostridiales;Lachnospiraceae; Pseudobutyrivibrio** |
| **26** | **Pseudobutyrivibrio ruminis** | **Pseudobutyrivibrio ruminis** | **Firmicutes; Clostridia; Clostridiales;Lachnospiraceae; Pseudobutyrivibrio** |
| **27** | **Pseudobutyrivibrio sp. LB2011** | **Pseudobutyrivibrio sp. LB2011** | **Firmicutes; Clostridia; Clostridiales;Lachnospiraceae; Pseudobutyrivibrio** |
| **28** | **Paenibacillus sp. JDR-2** | **Paenibacillus sp. JDR-2** | **Firmicutes; Bacilli; Bacillales; Paenibacillaceae;Paenibacillus** |
| **29** | **Roseburia faecis** | **Roseburia faecis** | **Firmicutes; Clostridia; Clostridiales;Lachnospiraceae; Roseburia** |
| **30** | **Pseudobutyrivibrio ruminis** | **Pseudobutyrivibrio ruminis** | **Firmicutes; Clostridia; Clostridiales;Lachnospiraceae; Pseudobutyrivibrio** |
| **31** | **Ruminococcaceae bacterium AB4001** | **Ruminococcaceae bacterium AB4001** | **Firmicutes; Clostridia; Clostridiales; Ruminococcaceae; unclassified Ruminococcaceae** |
| **32** | **Pseudobutyrivibrio sp. MD2005** | **Pseudobutyrivibrio sp. MD2005** | **Firmicutes; Clostridia; Clostridiales;Lachnospiraceae; Pseudobutyrivibrio** |
| **33** | **Pseudobutyrivibrio ruminis** | **Pseudobutyrivibrio ruminis** | **Firmicutes; Clostridia; Clostridiales;Lachnospiraceae; Pseudobutyrivibrio** |
| **34** | **Butyrivibrio fibrisolvens 16/4** | **Butyrivibrio fibrisolvens 16/4** | **Firmicutes; Clostridia; Clostridiales;Lachnospiraceae; Butyrivibrio** |
| **35** | **Lachnospiraceae bacterium AC2014** | **Lachnospiraceae bacterium AC2014** | **Firmicutes; Clostridia; Clostridiales; Lachnospiraceae** |
| **36** | **[Eubacterium] eligens** | **[Eubacterium] eligens** | **Firmicutes; Clostridia; Clostridiales; Eubacteriaceae; Eubacterium** |
| **37** | **Eubacterium eligens CAG:72** | **Eubacterium eligens CAG:72** | **Firmicutes; Clostridia; Clostridiales; Eubacteriaceae; Eubacterium;** |
| **38** | **Catonella morbi** | **Catonella morbi** | **Firmicutes; Clostridia; Clostridiales;Lachnospiraceae; Catonella** |
| **39** | **Eubacterium sp. CAG:86** | **Eubacterium sp. CAG:86** | **Firmicutes; Clostridia; Clostridiales; Eubacteriaceae; Eubacterium** |
| **40** | **Lachnospiraceae bacterium AC2029** | **Lachnospiraceae bacterium AC2029** | **Firmicutes; Clostridia; Clostridiales; Lachnospiraceae** |
| **41** | **Ruminococcaceae bacterium AB4001** | **Ruminococcaceae bacterium AB4001** | **Firmicutes; Clostridia; Clostridiales;Ruminococcaceae; unclassified Ruminococcaceae** |
| **42** | **Butyrivibrio fibrisolvens 16/4** | **Butyrivibrio fibrisolvens 16/4** | **Firmicutes; Clostridia; Clostridiales; Lachnospiraceae; Butyrivibrio** |
| **43** | **Ruminococcaceae bacterium AB4001** | **Ruminococcaceae bacterium AB4001** | **Firmicutes; Clostridia; Clostridiales; Ruminococcaceae; unclassified Ruminococcaceae** |
| **44** | **Butyrivibrio sp. AE2032** | **Butyrivibrio sp. AE2032** | **Firmicutes; Clostridia; Clostridiales; Lachnospiraceae; Butyrivibrio** |
| **45** | **Butyrivibrio sp. AE2032** | **Butyrivibrio sp. AE2032** | **Firmicutes; Clostridia; Clostridiales; Lachnospiraceae; Butyrivibrio** |
| **46** | **Butyrivibrio sp. AE2032** | **Butyrivibrio sp. AE2032** | **; Firmicutes; Clostridia; Clostridiales; Lachnospiraceae; Butyrivibrio** |
| **47** | **Butyrivibrio sp. AE2032** | **Butyrivibrio sp. AE2032** | **Firmicutes; Clostridia; Clostridiales; Lachnospiraceae; Butyrivibrio** |
| **48** | **Ruminococcaceae bacterium AB4001** | **Ruminococcaceae bacterium AB4001** | **Firmicutes; Clostridia; Clostridiales;Ruminococcaceae; unclassified Ruminococcaceae.** |
| **49** | **Ruminococcaceae bacterium AB4001** | **Ruminococcaceae bacterium AB4001** | **Firmicutes; Clostridia; Clostridiales;Ruminococcaceae; unclassified Ruminococcaceae** |
| **50** | **Lachnospiraceae bacterium P6B14** | **Lachnospiraceae bacterium P6B14** | **; Firmicutes; Clostridia; Clostridiales; Lachnospiraceae** |
| **51** | **Eubacterium saphenum** | **Eubacterium saphenum** | **Firmicutes; Clostridia; Clostridiales; Eubacteriaceae; Eubacterium** |
| **52** | **Butyrivibrio sp. AE2032** | **Butyrivibrio sp. AE2032** | **Firmicutes; Clostridia; Clostridiales; Lachnospiraceae; Butyrivibrio** |
| **53** | **Butyrivibrio sp. AE2032** | **Butyrivibrio sp. AE2032** | **Firmicutes; Clostridia; Clostridiales; Lachnospiraceae; Butyrivibrio** |
| **54** | **[Clostridium] saccharogumia** | **[Clostridium] saccharogumia** | **Firmicutes; Erysipelotrichia; Erysipelotrichales; Erysipelotrichaceae** |
| **55** | **Clostridiales bacterium VE202-01** | **Clostridiales bacterium VE202-01** | **Firmicutes; Clostridia; Clostridiales** |
| **56** | **Ruminococcaceae bacterium AB4001** | **Ruminococcaceae bacterium AB4001** | **Firmicutes; Clostridia; Clostridiales;Ruminococcaceae; unclassified Ruminococcaceae** |
| **57** | **Candidatus Stoquefichus massiliensis** | **Candidatus Stoquefichus massiliensis** | **Firmicutes; Erysipelotrichia; Erysipelotrichales; Erysipelotrichaceae; Candidatus Stoquefichus.** |
| **58** | **Coprobacillus** | **Coprobacillus** | **Firmicutes; Erysipelotrichia; Erysipelotrichales; Erysipelotrichaceae.** |
| **59** | **Candidatus Stoquefichus sp. SB1** | **Candidatus Stoquefichus sp. SB1** | **Firmicutes; Erysipelotrichia; Erysipelotrichales; Erysipelotrichaceae; Candidatus Stoquefichus.** |
| **60** | **Clostridium sp. CAG:58** | **Clostridium sp. CAG:58** | **Firmicutes; Clostridia; Clostridiales; Clostridiaceae;** |
| **61** | **Coprobacillus sp. 3_3_56FAA** | **Coprobacillus sp. 3_3_56FAA** | **; Firmicutes; Erysipelotrichia; Erysipelotrichales; Erysipelotrichaceae; Coprobacillus** |
| **62** | **Oribacterium parvum ACB1** | **Oribacterium parvum ACB1** | **Firmicutes; Clostridia; Clostridiales; Lachnospiraceae; Oribacterium** |
| **63** | **Erysipelatoclostridium ramosum DSM 1402 ([Clostridium] ramosum DSM 1402)** | **Erysipelatoclostridium ramosum DSM 1402 ([Clostridium] ramosum DSM 1402)** | **Firmicutes; Erysipelotrichia; Erysipelotrichales; Erysipelotrichaceae; Erysipelatoclostridium.** |
| **64** | **Erysipelotrichaceae** | **Erysipelotrichaceae** | **Firmicutes; Erysipelotrichia; Erysipelotrichales.** |
| **65** | **Oribacterium parvum** | **Oribacterium parvum** | **Firmicutes; Clostridia; Clostridiales; Lachnospiraceae; Oribacterium** |
| **66** | **Ruminococcus sp. CAG:55** | **Ruminococcus sp. CAG:55** | **Firmicutes; Clostridia; Clostridiales;Ruminococcaceae; Ruminococcus** |
| **67** | **[Ruminococcus] torques** | **[Ruminococcus] torques** | **Firmicutes; Clostridia; Clostridiales; Lachnospiraceae; Blautia.** |
| **68** | **[Ruminococcus] torques** | **[Ruminococcus] torques** | **Firmicutes; Clostridia; Clostridiales; Lachnospiraceae; Blautia** |
| **69** | **Ruminococcus torques L2-14** | **Ruminococcus torques L2-14** | **Firmicutes; Clostridia; Clostridiales; Lachnospiraceae; Blautia.** |
| **70** | **Oribacterium sp. FC2011** | **Oribacterium sp. FC2011** | **Firmicutes; Clostridia; Clostridiales; Lachnospiraceae; Oribacterium** |
| **71** | **[Ruminococcus] torques** | **[Ruminococcus] torques** | **; Firmicutes; Clostridia; Clostridiales; Lachnospiraceae; Blautia.** |
| **72** | **Oribacterium sp. NK2B42** | **Oribacterium sp. NK2B42** | **Firmicutes; Clostridia; Clostridiales; Lachnospiraceae; Oribacterium** |
| **73** | **Anaerofustis stercorihominis** | **Anaerofustis stercorihominis** | **Firmicutes; Clostridia; Clostridiales; Eubacteriaceae; Anaerofustis.** |
| **74** | **Clostridiales bacterium VE202-18** | **Clostridiales bacterium VE202-18** | **Firmicutes; Clostridia; Clostridiales** |
| **75** | **[Ruminococcus] torques** | **[Ruminococcus] torques** | **Firmicutes; Clostridia; Clostridiales; Lachnospiraceae; Blautia** |
| **76** | **[Ruminococcus] torques** | **[Ruminococcus] torques** | **Firmicutes; Clostridia; Clostridiales; Lachnospiraceae; Blautia** |
| **77** | **Coprobacillus sp. 3_3_56FAA** | **Coprobacillus sp. 3_3_56FAA** | **Firmicutes; Erysipelotrichia; Erysipelotrichales; Erysipelotrichaceae; Coprobacillus** |
| **78** | **Coprobacillus sp. 3_3_56FAA** | **Coprobacillus sp. 3_3_56FAA** | **Firmicutes; Erysipelotrichia; Erysipelotrichales; Erysipelotrichaceae; Coprobacillus** |
| **79** | **Coprobacillus sp. D7** | **Coprobacillus sp. D7** | **Firmicutes; Erysipelotrichia; Erysipelotrichales; Erysipelotrichaceae; Coprobacillus** |
| **80** | **Erysipelatoclostridium ramosum** | **Erysipelatoclostridium ramosum** | **Firmicutes; Erysipelotrichia; Erysipelotrichales; Erysipelotrichaceae; Erysipelatoclostridium.** |
| **81** | **[Clostridium] spiroforme** | **[Clostridium] spiroforme** | **Firmicutes; Erysipelotrichia; Erysipelotrichales; Erysipelotrichaceae; Erysipelatoclostridium.** |
| **82** | **Ruminococcus lactaris CC59_002D** | **Ruminococcus lactaris CC59_002D** | **Firmicutes; Clostridia; Clostridiales; Ruminococcaceae; Ruminococcus** |
| **83** | **Coprobacillus sp. 8_2_54BFAA** | **Coprobacillus sp. 8_2_54BFAA** | **Firmicutes; Erysipelotrichia; Erysipelotrichales; Erysipelotrichaceae; Coprobacillus.** |
| **84** | **Ruminococcus lactaris** | **Ruminococcus lactaris** | **Firmicutes; Clostridia; Clostridiales; Ruminococcaceae; Ruminococcus** |
| **85** | **uncultured bacterium (presumably a Firmicute)** | **uncultured bacterium (presumably a Firmicute)** | **environmental samples** |
| **86** | **Haloplasma contractile** | **Haloplasma contractile** | **Haloplasmatales; Haloplasmataceae; Haloplasma** |
| **87** | **Candidatus Stoquefichus sp. SB1** | **Candidatus Stoquefichus sp. SB1** | **Firmicutes; Erysipelotrichia; Erysipelotrichales; Erysipelotrichaceae; Candidatus Stoquefichus.** |
| **88** | **Coprobacillus** | **Coprobacillus** | **Firmicutes; Erysipelotrichia; Erysipelotrichales; Erysipelotrichaceae** |
| **89** | **Catenibacterium mitsuokai** | **Catenibacterium mitsuokai** | **Firmicutes; Erysipelotrichia; Erysipelotrichales; Erysipelotrichaceae; Catenibacterium** |
| **90** | **Firmicutes (Gram-positive bacteria)** | **Firmicutes (Gram-positive bacteria)** | **Firmicutes (Gram-positive bacterium), no further definition** |
| **91** | **Catenibacterium sp. CAG:290** | **Catenibacterium sp. CAG:290** | **Firmicutes; Erysipelotrichia; Erysipelotrichales; Erysipelotrichaceae; Catenibacterium** |
| **92** | **Clostridium sp. BR72** | **Clostridium sp. BR72** | **Firmicutes; Clostridia; Clostridiales; Clostridiaceae; Clostridium.** |
| **93** | **Clostridiaceae bacterium GM1** | **Clostridiaceae bacterium GM1** | **Firmicutes; Clostridia; Clostridiales; Clostridiaceae.** |
| **94** | **Clostridium sp. LF2** | **Clostridium sp. LF2** | **Firmicutes; Clostridia; Clostridiales; Clostridiaceae; Clostridium.** |
| **95** | **Ruminococcaceae bacterium GD1** | **Ruminococcaceae bacterium GD1** | **Firmicutes; Clostridia; Clostridiales; Ruminococcaceae; unclassified Ruminococcaceae** |
| **96** | **Clostridiales bacterium VE202-01** | **Clostridiales bacterium VE202-01** | **Firmicutes; Clostridia; Clostridiales.** |
| **97** | **Coprobacillus sp. 8_2_54BFAA** | **Coprobacillus sp. 8_2_54BFAA** | **Firmicutes; Erysipelotrichia; Erysipelotrichales; Erysipelotrichaceae; Coprobacillus** |
| **98** | **Clostridiales bacterium VE202-18** | **Clostridiales bacterium VE202-18** | **Firmicutes; Clostridia; Clostridiales** |
| **99** | **Coprobacillus sp. CAG:183** | **Coprobacillus sp. CAG:183** | **Firmicutes; Erysipelotrichia; Erysipelotrichales; Erysipelotrichaceae; Coprobacillus;** |
| **100** | **Fervidicella metallireducens** | **Fervidicella metallireducens** | **Firmicutes; Clostridia; Clostridiales; Clostridiaceae; Fervidicella.** |
